# Exogenous brassinosteroids alleviate calcium deficiency-induced tip-burn by maintaining cell wall structural stability and higher photosynthesis in mini Chinese Cabbage

**DOI:** 10.3389/fpls.2022.999051

**Published:** 2022-12-09

**Authors:** Yutong Li, Jizhong Ma, Xueqin Gao, Jianzhong Tie, Yue Wu, Zhongqi Tang, Linli Hu, Jihua Yu

**Affiliations:** ^1^ Gansu Provincial Key Laboratory of Aridland Crop Science, Gansu Agricultural University, Lanzhou, China; ^2^ College of Horticulture, Gansu Agricultural University, Lanzhou, China

**Keywords:** Tip-burn, deficiency calcium, brassinosteroids, cell wall structural, photosynthesis

## Abstract

Tip-burn has seriously affected the yield, quality and commodity value of mini Chinese cabbage. Calcium (Ca^2+^) deficiency is the main cause of tip-burn. In order to investigate whether exogenous brassinosteroids (BRs) can alleviate tip-burn induced by calcium (Ca^2+^) deficiency and its mechanism, in this study, Ca^2+^ deficiency in nutrient solution was used to induced tip-burn, and then distilled water and BRs were sprayed on leaves to observe the tip-burn incidence of mini Chinese cabbage. The tip-burn incidence and disease index, leaf area, fluorescence parameters (Fv/Fm, NPQ, qP andφPSII) and gas exchange parameters (Tr, Pn, Gs and Ci), pigment contents, cell wall components, mesophyll cell ultrastructure and the expression of genes related to chlorophyll degradation were measured. The results showed that exogenous BRs reduced the tip-burn incidence rate and disease index of mini Chinese cabbage, and the tip-burn incidence rate reached the highest on the ninth day after treatment. Exogenous BRs increased the contents of cellulose, hemifiber, water-soluble pectin in Ca^2+^ deficiency treated leaves, maintaining the stability of cell wall structure. In addition, BRs increased photosynthetic rate by increasing the activities of Ribulose-1,5-bisphosphate carboxylase/oxygenase (Rubisco) and fructose 1,6-bisphosphatase (FBPase) related to Calvin cycle, maintaining relatively complete chloroplast structure and higher chlorophyll content *via* down-regulating the expression of *BrPPH1* and *BrPAO1* genes related to chlorophyll degradation. In conclusion, exogenous BRs alleviated calcium deficiency-induced tip-burn by maintaining cell wall structural stability and higher photosynthesis.

## Introduction

Mini Chinese cabbage (*Brassica rapa* L. *ssp. pekinensis*) is one of the main cultivated summer vegetables in plateaus, as well as an important export vegetable in China. In recent years, with global climate change, tip-burn has become exceptionally common in mini Chinese cabbage, which is generally considered a calcium-associated physiological disorder (Thibodeau, 1969; Bangerth, 1979), and negatively affect the quality and yield of mini Chinese cabbage (Everaarts and Blom-Zandstra, 2001). Tip-burn mostly occurs at the rosette stage, but can also occur at the seedling stage if the growth environment is not suitable or if the variety has weak resistance to the disease. The occurrence of tip-burn shows that the edges of plant leaves shrink, chlorosis and finally dry into brown paper, which it causes the structure and function destruction of cell membrane and cell wall, leading to meristem necrosis, and even the infection of pathogenic bacteria in the late stage of the disease (Su et al., 2016), which seriously affects the appearance quality, taste and commodity value of vegetables (Saure, 1998). Therefore, it is necessary to find an effective strategy to prevent and control the occurrence of tip-burn key problems to be solved urgently in mini Chinese cabbage production.

Calcium (Ca^2+^) is an important regulator of plant growth and development, which will have a great impact on plant quality (Lecourieux et al., 2006), as well as, it is a component of plant cell wall and cell membrane, maintaining the stability of cell wall structure and normal physiological functions in the process of maintaining plants from abiotic stresses (Zhu, 2016). Calcium is also an important structural, metabolic and signaling element (Tuteja, 2009). The physiological function of Ca^2+^ is realized through the orderly transport across cell membrane mediated by Ca^2+^ osmotic ion channel, Ca^2+^-ATPase and Ca^2+^/H^+^ exchanger (Straltsova et al., 2015). The occurrence of tip-burn is particularly serious in the facility environment (Lee et al., 2013). This may occur because facility conditions promote plant growth and faster growth rate, the formation and expansion of new tissue cell walls require large amounts of calcium, and the calcium demand becomes much higher than the actual absorption, thus increasing the incidence of tip-burn (Borkowski et al., 2016). Although its pathogenesis is complex and controversial, it is generally accepted that the inability of plants to provide sufficient calcium for rapidly developing leaves is the main reason for tip-burn (Kuronuma et al., 2019; Su et al., 2019; Wang et al., 2019).

In 1970, Mitchell and his team discovered a substance in the extract of rapeseed pollen that promoted plant cell division and elongation (Mitchell et al., 1970), subsequently Grove et al. (1979) defined this substance as brassinosteroid (BRs). In 1980, Fung and Siddall (1980) used their previous studies as a guide to successfully synthesize BRs for the first time. BRs constitute the sixth major class of plant hormones, and they are also recognized as highly efficient, broad-spectrum, non-toxic plant growth regulators, with strong penetration and fast internal absorption (Kurepin et al., 2016). BRs has concentration dose effect, at very low concentrations, they can significantly increase the growth of plants and regulate their various developmental processes (J Ahammed et al., 2015; Ahammed et al., 2020), including root and hypocotyl elongation, seed germination, flowering, and fruiting. In addition, they can mediate various stimuli in response to the environment, improve abiotic stress resistance in crops such as water stress (Li and Feng, 2011), high temperature stress (Cao and Hua, 2008),chilling damage (Sun et al., 2020), salinity stress (Su et al., 2020), heavy metal stress (Bajguz and Botany, 2010), and drought stress (Naveen et al., 2021), and significantly reduce the occurrence of diseases such as *Botrytis cinerea* infection (Li et al., 2020), rice blast and bacterial blight diseases (Nakashita et al., 2003). Fariduddin et al. (2009) showed that there is a concentration effect of BRs application in plants, and that the appropriate concentration BRs enhanced chlorophyll content and photo-synthetic efficiency in cucumbers. Spraying BRs at appropriate concentrations on tomato crops can enhance the activity of RuBP carboxylation/oxygenase (Rubisco), increase stomatal opening of tomato leaves and improve photosynthesis (Ogweno et al., 2008). Yamamoto et al. (1997) demonstrated the role of BRs on secondary cell wall formation and cell death during tracheary element differentiation in *Zinnia* system.

The above literatures showed that BR had certain effects on plant growth and development (photosynthetic characteristics, cell wall homeostasis, as so on) as well as various stresses. However, there are few reports on the relationship between BR and Ca^2+^ deficiency induced tip-burn. Therefore, we explored the effects of BRs on the growth and development, photosystem II (Fv/Fm, qP and Y (II)) and photosynthetic segment content, cell wall components of mini Chinese cabbage during Ca^2+^ deficiency induced tip-burn in this study. The results revealed the mitigation mechanism of BRs on the Ca^2+^ deficiency induced tip-burn in mini Chinese cabbage, which provided a new strategy and theoretical basis for overcoming the occurrence of tip-burn in mini Chinese cabbage cultivation.

## Materials and methods

### Plant material and growth conditions

The seeds of mini Chinese cabbage (*Brassica pekinensis* cv. Qiu Yu Huang) were surface sterilized in 5% sodium hypochlorite for 10 min, placed in a wet towel covered with double-layer filter paper, and then germinated for 2 d at 25°C in the dark. The germinated seeds were transplanted into planting cotton (30 cm × 20 cm × 3 cm) and placed at 25 ± 1°C and 17 ± 1°C (day and night, respectively) for 12 d with a 14 h photoperiod (photosynthetically active radiation = 200 µmol m^-2^ s^-1^) in an incubator. At the two-leaf stage, the uniform growth seedlings were transplanted into a hydroponic box (20 cm × 11 cm × 8 cm) filled with 700 mL improved Hoagland’s solution at pH 6.0 for 12 d of cultivation and each hydroponic box contained two seedlings. Analytical grade chemicals used in this study were obtained from Chinese companies [Sinopharm Chemical Reagent Co., Ltd)].

### Experimental design

At the five-leaf stage, the plants were transplanted to different formulae of Hoagland’s nutrient solution (Wang et al., 2019), improved Hoagland’s solution and Hoagland’s nutrient solution without Ca^2+^. In preliminary experiment, the suitable concentration of BR (0.5µM) was screened out, which could reduce the incidence rate and disease index of tip-burn in mini Chinese cabbage seedlings. In the present experiment, the seedlings were treated as follows:

(a) (+Ca) Con + H_2_O: Normal Ca^2+^-containing nutrient solution + foliar spray distilled water.
(b) (+Ca) Con + 0.5 µM BR: Normal Ca^2+^-containing nutrient solution + foliar spray 0.5 µM BR.
(c) (−Ca) Con + H_2_O: Ca^2+^-free nutrient solution + foliar spray distilled water.
(d) (−Ca) Con + 0.5 µM BR: Ca^2+^-free nutrient solution + foliar spray 0.5 µM BR.

The seedlings were sprayed with BRs every 2 d and the amount of BRs (0.3-0.5 mL) was used to wet each leaf, subject to no dripping. The symptoms of tip-burn initially appeared at 3 d after treatment and the seedlings were treated for 12 d. For each treatment, 1 cm of leaf margin tissue was obtained from three individual plants and equally homogenized as one sample, which was immediately frozen with liquid nitrogen and stored in an ultra-low temperature refrigerator at −80°C. Each treatment had three biological replicates.

### Determination of incidence rate and disease index of tip-burn

Whole leaves were scored using an arbitrary tip-burn severity index, with a ranking from 0-9. The classified criterion of tip-burn of mini Chinese cabbage was presented as follow:
0: Asymptomatic.
0.5: Only small spots on the edge of true leaves.
1: The edge of a leaf is chlorosis.
3: The edges of two leaves are chlorosis and wrinkled.
5: The edges of more than two leaves are slightly tip-burn, and the tip-burn area accounts for less than 25% of the leaves.
7: The edges of more than two leaves are moderately tip-burn, and the tip-burn area accounts for 25%–50% of the leaf area.
9: The edges of more than two leaves are severely tip-burn or the whole plant dies, and the tip-burn area accounts for more than 50% of the leaf area.
The severity of tip-burn in each plant was defined as follows:
Tip-burn disease index = Σ {(severity index × leaf number)/total number of leaves per pot}× 100 (Corriveau et al., 2012)
The incidence of tip-burn was expressed as the percentage of plants exhibiting tip-burn symptoms in all cultivation plants.

### Determination of leaf area

The leaves of mini Chinese cabbage on the ninth day of treatment were selected as samples. The functional leaves near the same growth point (The fourth functional leaf of mini Chinese cabbage seedlings counted from inside to outside), determination using root scanner (win rhizo Pro la2400, Canada).

### Determination of cell wall components

The cell wall components (cellulose, hemicellulose, water-soluble pectin) were determined according to the methods of Fishman et al. (1993); Brummell et al. (2004) and Thygesen et al. (2005), respectively.

### Determination of chlorophyll fluorescence parameters

After 30 min of full dark reaction, the chlorophyll fluorescence parameters of seedlings on the ninth day of treatment were measured by the Chl fluorescence imaging (imaging WinGigE, Walz, Effeltrich, Germany) (Hu et al., 2017). The calculation formula is as follows:


Fv/Fm=(Fm−Fo)/Fm

φPSⅡ= Δ F/Fm'=(Fm'−F)/Fm'=(Fm'−Fs)/Fm'

qP=(Fm'−Fs)/Fv'=1−(Fs−Fo')/(Fm'−Fo')

NPQ=(Fm−Fm')/Fm'=Fm/Fm'−1;

### Determination of photosynthetic gas exchange

The fourth functional leaf of mini Chinese cabbage seedlings counted from inside to outside on the ninth day after treatment was selected to measure gas exchange parameters with photosynthetic apparatus (CIRAS-2, UK).

### Determination of RUBP carboxylation/oxygenase activity and fructose 1,6-bisphosphatase activity

RuBP carboxylation/oxygenase (Rubisco) and fructose 1,6-bisphosphatase (FBPase) activity detection kits (Suzhou Keming Biotechnology Co., Ltd., China) were used to determine the activities of Rubisco and FBPase in mini Chinese cabbage leaves, respectively. Refer to the instructions of the kit for specific operation steps.

### Ultrastructure of mesophyll cells

The fresh leaves of mini Chinese cabbage treated for the ninth day were used as samples. Take about 1cm^2^ small pieces with a punch, fix them in 3% glutaraldehyde, and conduct vacuum pumping to sink the leaf tissue to the bottom. Rinse with 0.1 M phosphate buffer (pH 7.4) for 3 times, soak for 10 min (elute malondialdehyde), and place at 4 °C for 24 h. Then soak it in 1% Russian acid for 5 h, rinse it with phosphoric acid buffer for 3 times, then dehydrate it with ethanol of different concentration gradient, and then wash it with acetone, then penetrate the acetone and embed it into EPON812 epoxy resin for embedding. Ultrathin sample sections were cut on the ultramicro body (LeicaEMUC6 ultramicro, Japan), and then stained with uranyl acetate and lead citrate for 15 min. The ultrathin sections of mini Chinese cabbage leaves were observed and photographed by transmission electron microscope (TEM, JoelJem-1230, Japan).

### Determination of photosynthetic pigment content

The leaves of mini Chinese cabbage seedlings on the ninth day of treatment were randomly perforated and sampled from the same part leaves, then put into a test tube filled with 80% acetone and placed in the dark for 24 h. The absorbance values of the extract at 665, 649 and 470 nm were measured by UV-1780 spectrophotometer (Shimadzu, Japan), and the contents of chlorophyll a (Chla), chlorophyll b (Chlb), total chlorophyll (Chla+b) and carotenoid (Car) were calculated as described by Anwar et al. (2018).

### RNA extraction and RT-PCR

Total RNA was isolated from 100 mg (fresh weight) of excised mini Chinese cabbage seedling leaves by grinding with a mortar and pestle in liquid nitrogen to obtain a fine paste using TaKaRa reagent (TaKaRa Bio, Japan) according to the manufacturer’s instructions. Each treatment was performed in triplicate. Primers designed for genes and reference genes were listed in **Table 1**. Each reaction (20 µL total volume) consisted of SYBR Premix Ex Taq II (10 µL), diluted cDNA (2 µL), forward and reverse primers (0.8 µL each), and nuclease-free water (6.4 µL). PCR amplification conditions were as follows: 1 min at 95°C followed by 40 cycles of 5 s at 95°C and 20 s at 60°C with data collection at the annealing step. After 40 cycles, the dissociation/melting curve stage at 95°C, 65°C for 15 s, 95°C for 0 s, and 50°C for 30 s. The relative quantification of mRNA was based on the method described by Livak and Schmittgen (2001). The expression level relative to the control for each sample was expressed as 2^-ΔΔCt^.

**Table 1:** Gene specific primers used for real-time PCR.

Genes	Accession number	Primer name	Primer sequence
*BrPAO1*	Bra006210.1	Primer F	GGCGTCGGTGGTAAAGAGTCTAAC
		Primer R	CGAGTCAGCAGCGAATCCAGT
*BrPPH1*	AC189212.2	Primer F	TTCCTCCTCCGTCACCTCCATTG
		Primer R	AGGCTGAAGCATTGGCGAATCC
*BrACTIN*	JN120480	Primer F	CCAGGAATCGCTGACCGTAT
		Primer R	CTGTTGGAAAGTGCTGAGGGA

### Statistical analysis

Data analysis was performed using Duncan’s multiple tests (*P*< 0.05) using SPSS software (version 19.0; IBM SPSS, Chicago, USA). Each experiment was performed in triplicate and the data are expressed as mean values ± SE (n=3).

## Result

### Effects of exogenous BRs on tip-burn incidence rate, disease index and leaf area size of mini Chinese cabbage induced by Ca^2+^ deficiency

**Figure 1:**
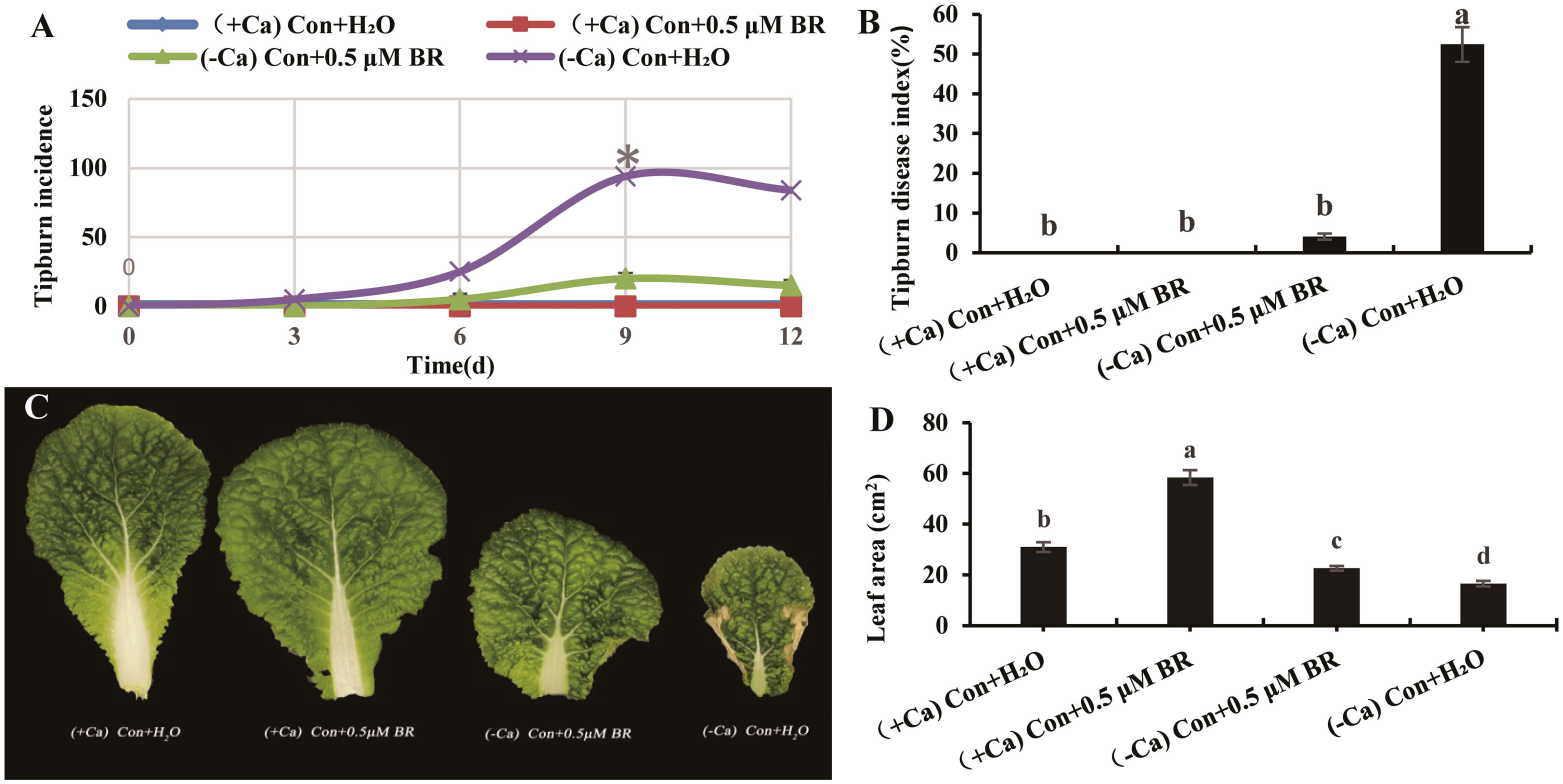
Effects of exogenous BRs on tip-burn incidence rate, disease index and leaf area of mini Chinese cabbage induced by Ca^2+^ deficiency. **(A)** the incidence rate of tip-burn at 0, 3, 6, 9 and 12 d after treatment. **(B)** the disease index of tip-burn on 9th day; **(C)** the fourth functional leaf counted from inside to outside of mini Chinese Cabbage seedlings; **(D)** Leaf area. The different letters above the bars showed significantly different among treatments according to Duncan’s multiple test (*P*< 0.05).

As shown in **Figure 1A**, the tip-burn incidence rates of seedling grown under different treatments from 0 d to 12 d were recorded every 3 days. The tip-burn incidence rate of seedling treated with (-Ca) Con+H_2_O increased continuously from 0 d to 9 d, and reached the maximum on 9th day. On the 12th day, the number of seedlings died from tip-burn, which led to a decrease in the incidence rate. Therefore, the 9th day was chosen for the following indexes measurements.

The tip-burn disease index on the ninth day between different treatments was shown in **Figure 1B**. Ca^2+^ deficiency significantly increased the tip-burn disease index, while foliar spray 0.5 µM BR significantly reduced the disease index of tip-burn by 92.36% compared to (- Ca) Con + H_2_O. Therefore, it can be speculated that exogenous BRs (0.5 µM) can reduce the tip-burn incidence rate and disease index of mini Chinese cabbage caused by Ca^2+^deficiency (**Figures 1A, B**). As shown in **Figures 1C, D**, the tip-burn of mini Chinese cabbage seriously affects the leaf area. Compared with (- Ca) Con + H_2_O treatment, exogenous spraying BRs significantly increased the leaf area by 36.38%. Similarly, under (+ Ca) nutrient solution treatment, exogenous BRs significantly increased the leaf area by 88.2%, compared with the treatment of exogenous spraying distilled water. In conclusion, exogenous BRs could significantly reduce the incidence rate and disease index of tip-burn induced by Ca^2+^ deficiency, as well as promote the leaf area of cabbage.

### Effects of exogenous BRs on cell wall components of mini Chinese cabbage seedling

**Figure 2:**
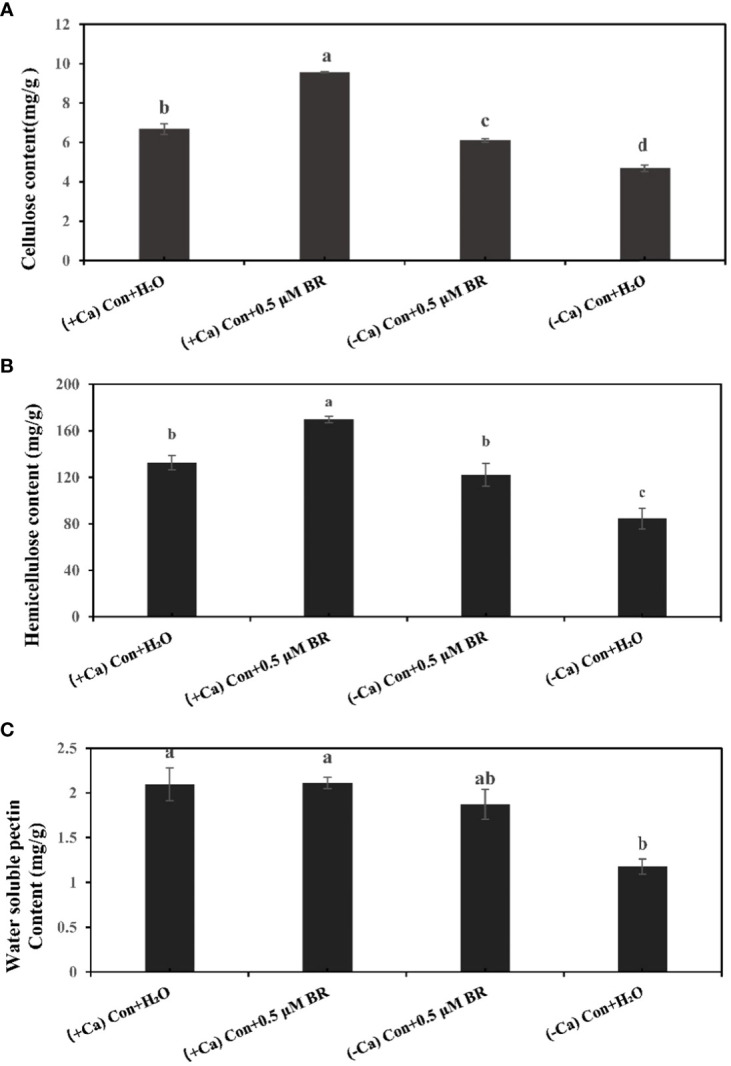
Effect of exogenous BRs on cell wall components of mini Chinese cabbage leaves. **(A)** cellulose. **(B)** hemicellulose. **(C)** water-soluble pectin. The different letters above the bars showed significantly different among treatments according to Duncan’s multiple test (*P<* 0.05).

Plant cell wall as the first defense system will be affected after stress. The content of cell wall components will change with the time of stress. Compared with (+ Ca) nutrient solution culture, Ca^2+^ deficiency induced tip-burn significantly reduces the contents of cellulose (**Figure 2A**), hemicellulose (**Figure 2B**) and water soluble pectin (**Figure 2C**), which proves that tip-burn seriously destroys the components of cell wall, destroys the structure of cell wall, weakens the defense system of cell wall and hinders plant growth. Under the culture condition of (+ Ca), Compared with the treatment of spraying distilled water, spraying BRs increased the contents of cellulose and hemicellulose by 43.1% and 27.8%, respectively. Similarly, under (- Ca) environmental conditions, compared with the treatment of spraying distilled water, spraying exogenous BRs significantly increased the contents of cellulose, hemicellulose and pectin by 30.4%, 44.68% and 59.8%, respectively. In conclusion, exogenous BRs enhanced the contents of cell wall components, maintaining cell wall structure stability in calcium deficiency leaves.

### Effects of exogenous BRs on chlorophyll fluorescence parameters and photosynthetic gas exchange of mini Chinese cabbage

As shown in **Table 2**, compared with calcium exist treatment, calcium deficiency significantly reduced the Fv/Fm, NPQ, qP, and φPSII values of mini Chinese Cabbage seedlings. After exogenous spraying BRs, the above values increased significantly. The excitation pressure of PSII (1-qP) and the excess excitation energy of reaction center [(1-qP)/NPQ] significantly increased under calcium deficiency condition, while leaf spraying BRs significantly decreased the two values. The above results suggested that BRs reduced the excess excitation energy of PSII through heat dissipation, so as to reduce the excitation pressure of leaf reaction center, to resist the photoinhibition induced by calcium deficiency in mini Chinese cabbage.

**Table 2:** Effect of exogenous BRs on chlorophyll fluorescence parameters of mini Chinese cabbage.

Treatment	Fv/Fm	NPQ	qP	φPSⅡ	1-qP	(1-qP)/NPQ
**(+Ca)Con+H_2_O**	0.710 ± 0.01a	0.208 ± 0.01b	0.870 ± 0.01a	0.518 ± 0.01ab	0.129 ± 0.01c	0.623 ± 0.04c
**(+Ca)Con+0.5μMBR**	0.733 ± 0.01a	0.279 ± 0.02a	0.899 ± 0.01a	0.553 ± 0.002a	0.100 ± 0.01c	0.363 ± 0.21c
**(-Ca)Con+0.5μMBR**	0.618 ± 0.04b	0.182 ± 0.01b	0.769 ± 0.01b	0.426 ± 0.06b	0.230 ± 0.01b	1.276 ± 0.30b
**(-Ca)Con+ H_2_O**	0.474 ± 0.03c	0.115 ± 0.02c	0.585 ± 0.04c	0.267 ± 0.04c	0.414 ± 0.04a	4.085 ± 0.62a

* The different lowercase letters in the same column showed significantly different among treatments according to Duncan’s multiple test (P< 0.05).

Under normal nutrient solution condition, exogenous BRs significantly increased the net photosynthetic rate (Pn), stomatal conductance (Gs) and transpiration rate (Tr) values. However, calcium deficiency significantly decreased these values, while foliar spraying BRs significantly reversed the negative effects of calcium deficiency on these parameters, which increased Tr, Gs and Pn by 27.4%, 34% and 170.9%, respectively (**Table 3**). Moreover, calcium deficiency significantly increased the intercellular carbon dioxide concentration (Ci), while exogenous BRs significantly decreased Ci under calcium deficiency conditions (**Table 3**). In conclusion, exogenous BRs can alleviate calcium deficiency induced tip-burn by maintaining higher photosynthesis.

**Table 3:** Effect of exogenous BRs on photosynthetic gas exchange in mini Chinese cabbage.

Treatments	Ci/ (μmol·mol^-1^)	Tr/ (mmol·m^-2^·s^-1^)	Gs/ (mmol·m^-2^·s^-1^)	Pn/ (μmol·m^-2^·s^-1^)
**(+Ca) Con+H_2_O**	379.33 ± 8.21b	2.5 ± 0.17b	125.67 ± 12.19b	3.07 ± 0.81b
**(+Ca) Con+0.5 μMBR**	389 ± 5.13b	3.37 ± 0.58a	203.67 ± 45.04a	4.43 ± 0.32a
**(-Ca) Con+0.5 μMBR**	400.66 ± 9.53b	2.46 ± 0.03b	130 ± 4.35b	2.47 ± 0.37b
**(-Ca) Con+ H_2_O**	520.33 ± 1.30a	1.93 ± 0.03c	97 ± 2.08c	0.91 ± 0.28c

*Ci, Internal CO_2_ concentration; Tr, Transpiration rate; Gs, Stomatal conductance; Pn, Net photosynthesis rate;

Data are expressed as the mean ± standard error (SE) of three replicates. Different lowercase letters in the same column indicated significant differences at p< 0.05, according to Duncan’s test.

### Effects of exogenous BRs on the activities of Rubisco and FBPase in mini Chinese cabbage leaves

**Figure 3:**
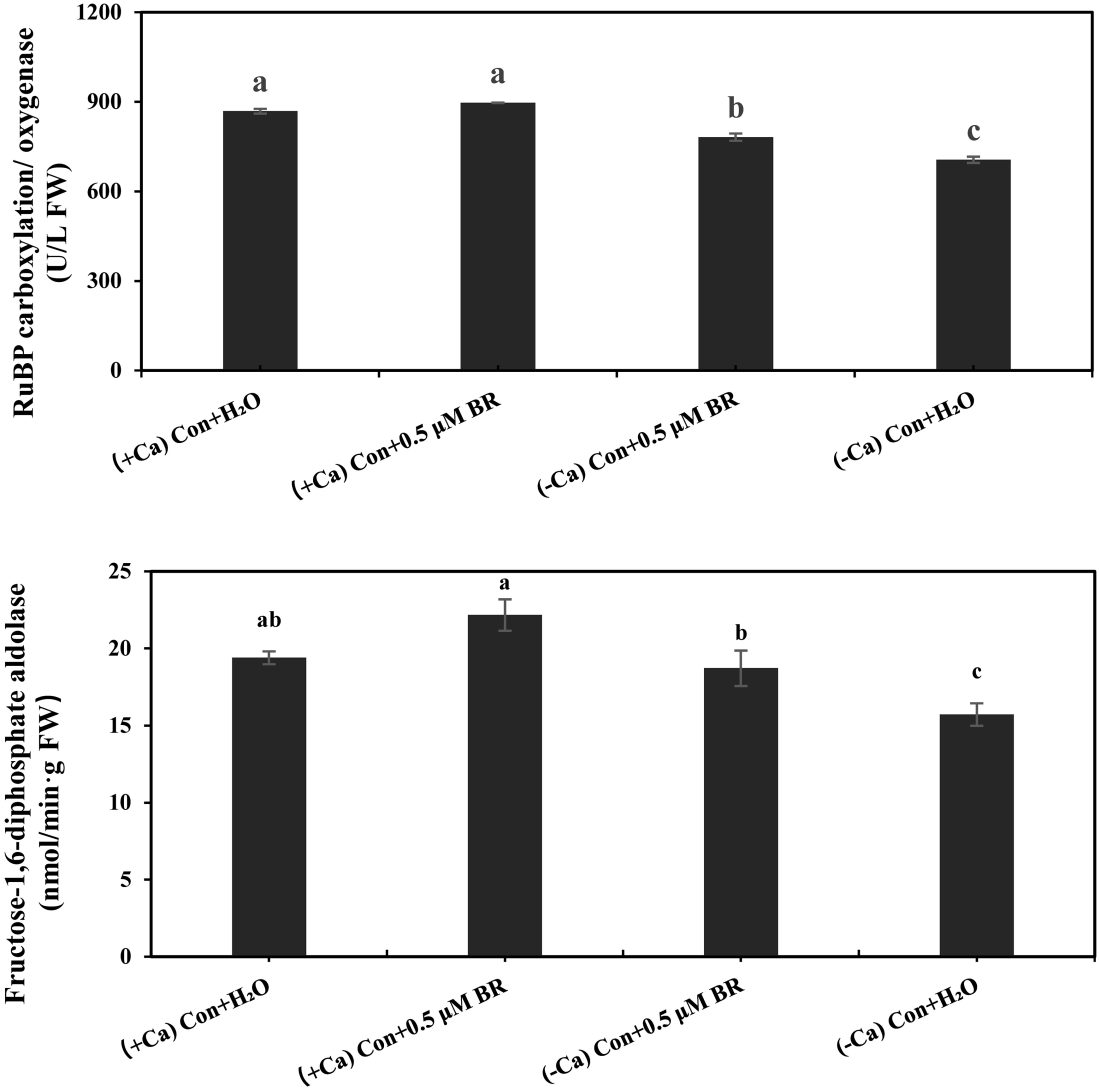
Effects of exogenous BRs on the activities of Rubisco and FBPase in mini Chinese cabbage leaves. Data are presented as mean ± SE (n=3). The different letters above the bars showed significantly different among treatments according to Duncan’s multiple test (*P<* 0.05).

As shown in **Figure 3**, Compared with (+ Ca) treatment, (- Ca) significantly inhibited the activities of Rubisco and FBPase by 187.6% and 18.98%, respectively. Exogenous spraying of BRs significantly increased the activity of photosynthetic enzyme, compared with (- Ca) Con + H_2_O treatment, the (- Ca) Con + 0.5 μM BR treatment significantly increased the activity of Rubisco and FBPase by 107.7% and 19.17%, respectively. In conclusion, exogenous BRs enhanced the photosynthesis of leaves by promoting the activity of Rubisco and FBPase.

### Effect of exogenous BRs on chloroplast ultrastructure of mini Chinese cabbage

**Figure 4:**
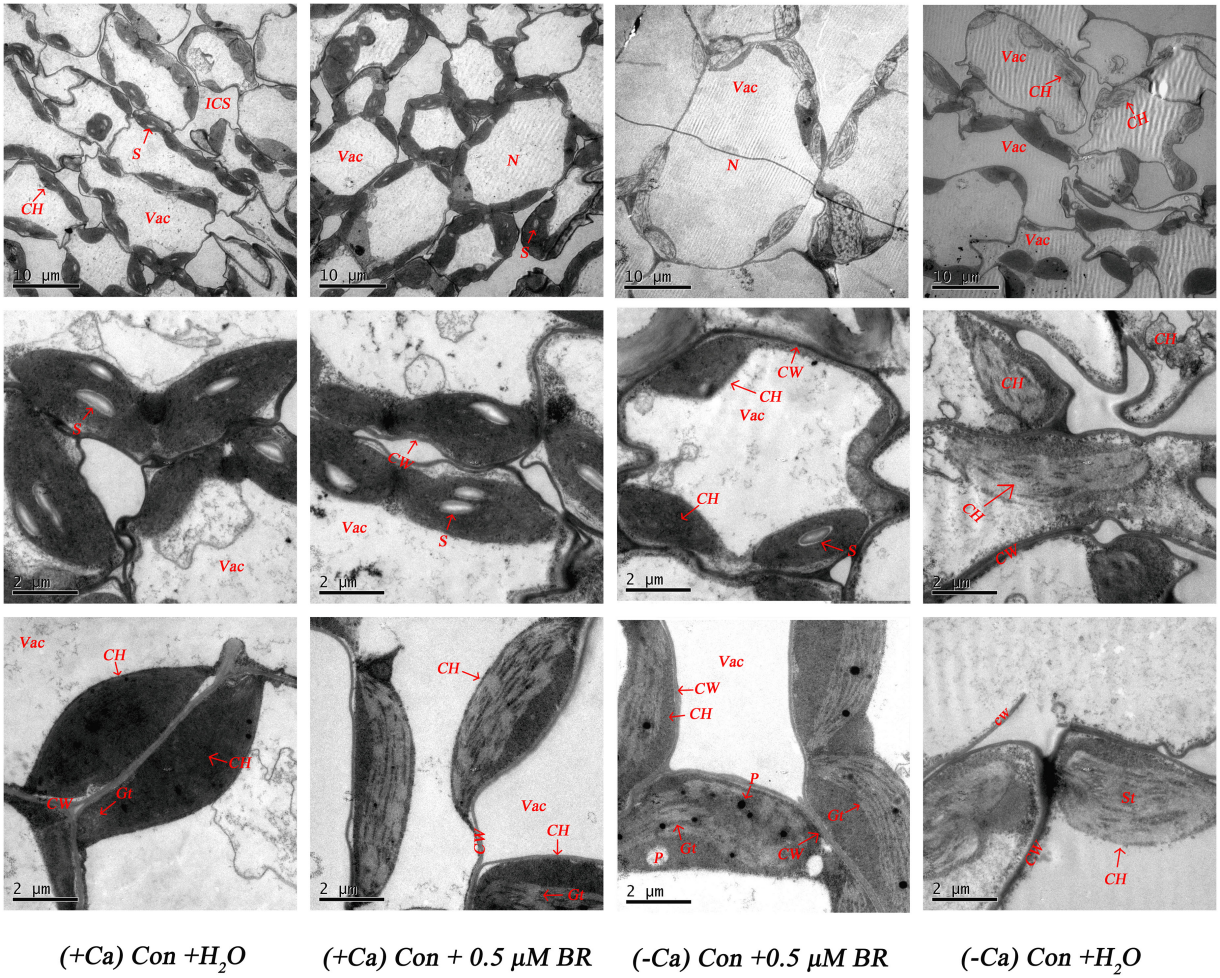
Effect of exogenous BRs on mesophyll cells ultrastructure of mini Chinese cabbage. CW, cell wall; Vac, vacuole; ICS, intercellular space; S, starch granule; ch, chloroplast; N, cell; P, plastid globule; OG, osmiophilic granule; ST, stromal lamella. The scale of chloroplasts was 10 μ m and 2 μ m.

As shown in **Figure 4**, in normal nutrient solution (+ Ca) treated leaf, most cells had complete structure. In individual cells, chloroplasts are arranged neatly close to the cell wall in the shape of spindle. The edge of chloroplasts is smooth, the thylakoid matrix lamella is clearly visible, arranged tightly and orderly, there are many and obvious starch particles, and osmiophilic particles do not appear. Compared with the treatment of (+ Ca) culture, Ca^2+^ deficiency reduced the number of chloroplasts, the stromal lamella was chaotic. The cell wall was prone to plasmolysis, the starch granule in the cell were reduced, the osmiophilic granule were increased, the chloroplasts were not close to the cell wall, the membrane structure was fuzzy, and some chloroplasts even differentiated into irregular structures. However, in (-Ca) Con+0.5μM BR treated leaf, the cell wall did not separate, the cell membrane structure and the chloroplast structure were relatively intact, and it have a relatively good thylakoid structure. There were less osmiophilic granule in the chloroplast, but also a small amount of starch granule. In conclusion, exogenous BRs had a certain alleviating effect on the damage of chloroplast ultrastructure caused by calcium deficiency.

### Effect of exogenous BRs on chlorophyll content of mini Chinese cabbage seeding

**Figure 5:**
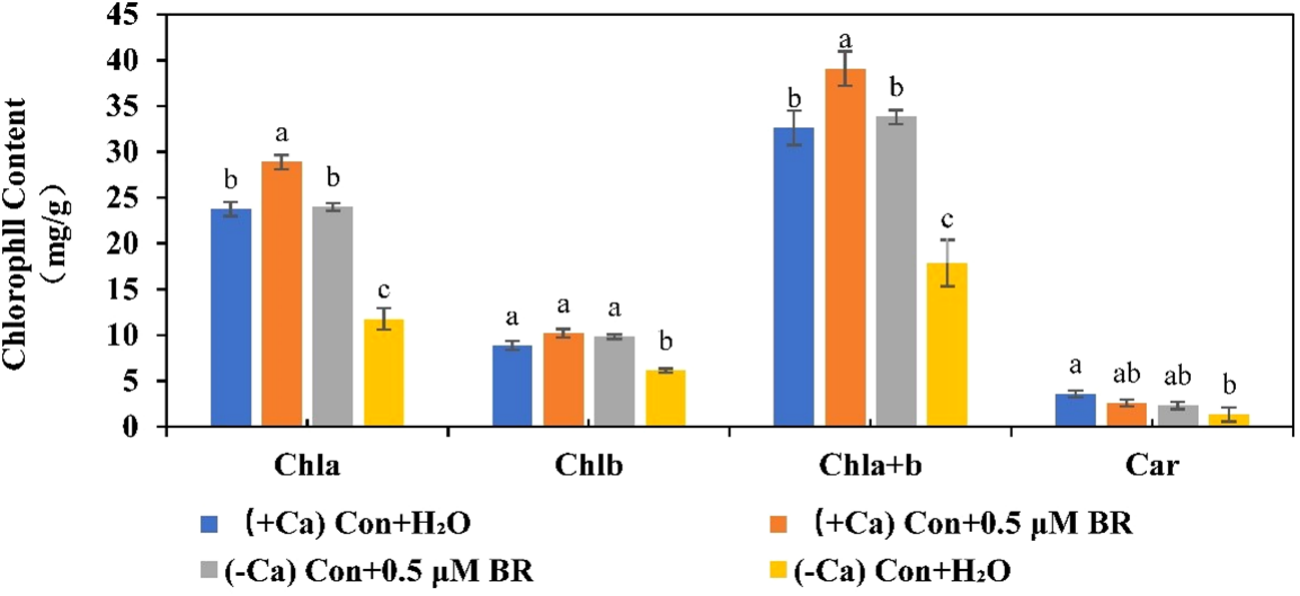
Effect of exogenous BRs on chlorophyll content in mini Chinese cabbage. Data are presented as mean ± SE of three replicates. The different letters above the bars showed significantly different among treatments according to Duncan’s multiple test (*P<* 0.05).

Photosynthetic pigments are involved in absorbing and transmitting light energy or causing primary photochemical reactions in photosynthesis. As shown in **Figure 5**, in (+ Ca) nutrient treated leaf, exogenous BRs increased the content of Chla, Chlb, and Chla+b. However, when plants were exposed to the calcium deficiency nutrient solution, the contents of Chla, Chlb, Chla+b and Car can be significantly reduced by 50.5%, 30.9%, 45.2% and 63.02%, respectively. while Chla, Chlb and Chla+b contents of seedlings sprayed with exogenous BRs increased significantly compared with those sprayed with distilled water. As well as, exogenous BRs treatment increased the content of Car, but it was not significant, indicating that the effect of BRs on the content of Car was also regulated by other factors. In conclusion, these results suggested that exogenous BRs alleviated calcium deficiency induced tip-burn by increasing photosynthetic pigment contents, thus delaying calcium deficiency caused leaf senescence and maintaining higher photosynthesis.

### Effect of exogenous BRs on the expression of chloroplast degradation related genes in mini Chinese cabbage

**Figure 6:**
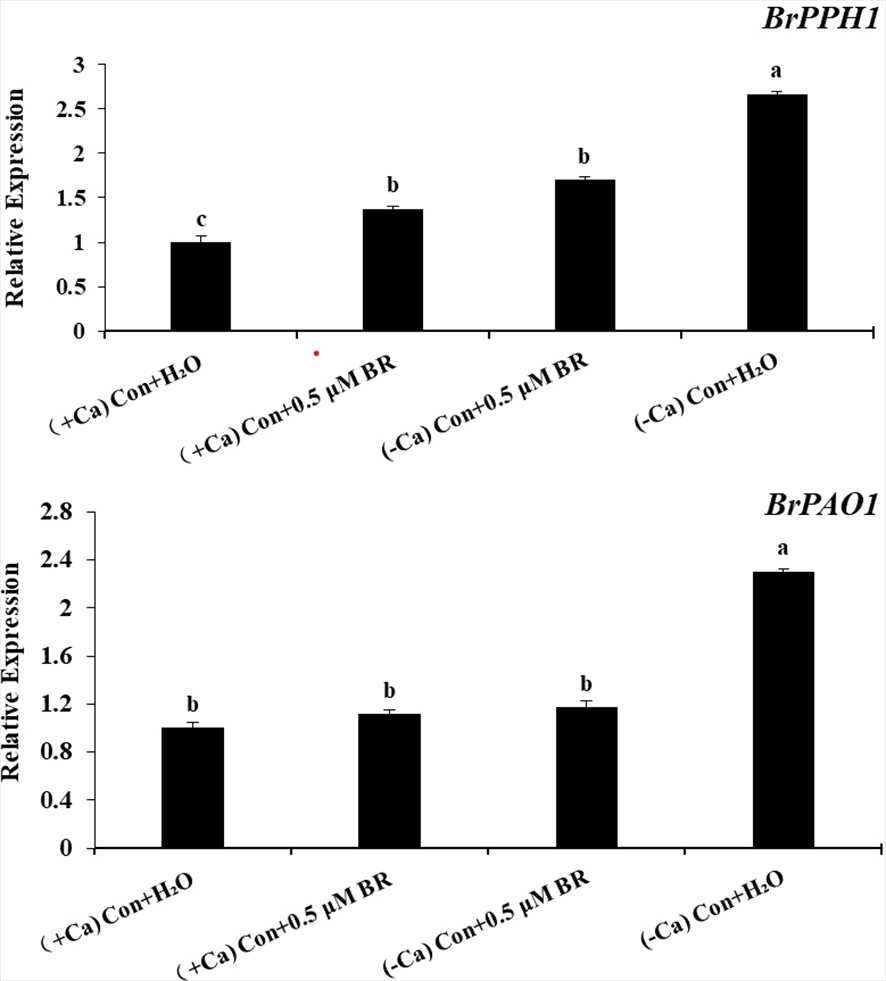
Effect of exogenous BRs on the expression levels of genes related to chloroplast degradation in mini Chinese cabbage leaves. The relative expression level of BrPPH1 and BrPAO1 on ninth day were expressed as mean ± SE (n =3). The different letters above the bars showed significantly different among treatments according to Duncan’s multiple test (*P*< 0.05).

As shown in **Figure 6**, compared with (+ Ca) treatment, calcium deficiency significantly up-regulated the gene expression of *BrPPH1* and *BrPAO1*, which proved that mini Chinese cabbage with tip-burn could promote the degradation of leaf chlorophyll and accelerate leaf aging. Compared with (- Ca) Con + H_2_O treatment, exogenous BRs could down-regulate the expression of *BrPPH1* and *BrPAO1* by 35.97% and 49.02%, respectively. In conclusion, exogenous BRs can inhibit the expression of chlorophyll degradation related genes in mini Chinese cabbage leaves, increase the content of chlorophyll, enhance the photosynthesis and promote the recovery of normal growth of mini Chinese cabbage.

## Discussion

Calcium deficiency is the main factor inducing the occurrence of tip-burn. Meanwhile, tip-burn is the main disease restricting the yield and quality of mini Chinese cabbage (Aloni, 1986). How to alleviate and prevent the occurrence of tip-burn is an urgent problem to be solved in the cultivation of cabbage. Since the plant hormone brassinosteroids (BR) was discovered in the extract of rape pollen by Mitchell et al. (1970) and Grove et al. (1979), more and more experts have found that it can promote various processes of plant growth and development (Aghdam et al., 2014; Kurepin et al., 2016). In addition, BRs also has a certain relationship with plant disease resistance (Nakashita et al., 2003; Ding et al., 2021). In this experiment, Ca^2+^ deficiency in nutrient solution was used to induced the occurrence of tip-burn, and exogenous spraying of a certain concentration of BRs was used to alleviate the occurrence of tip-burn. We found that 0.5 μM BR reduced the tip-burn incidence rate and disease index of mini Chinese cabbage (**Figures 1A and B**). Among the 12 d of treatment, we recorded the incidence rate and disease index daily. The data showed that the incidence rate reached maximum values on the ninth day after treatment (**Figure 1A**). Therefore, the samples on 9^th^ day after treatment were used to measure the following indices. Leaf area is related to the efficiency of leaf photosynthesis. It had been reported that the plant photosynthetic rate and leaf area size were reduced under external abiotic stress conditions such as water, salinity stress, and drought (He and Cramer, 1996; Dalirie et al., 2010; Slabbert and Krüger, 2014). The leaf area of mini Chinese cabbage in which tip-burn occurred was significantly reduced, while exogenous BRs application could increase leaf area and maintain normal plant growth (**Figures 1C, D**). This finding is consistent with those of the study of Ali et al. (2021). Plant cell wall is involved in maintaining a certain shape of cells, enhancing the mechanical strength of cells, and is also related to the physiological activities of cells. When plants are subjected to stress, the cell wall is the first to undergo changes, and the content of cell wall components decreases significantly (Braidwood et al., 2014; Tenhaken, 2015). In our study, calcium deficiency significantly decreased the levels of cellulose, hemicellulose and pectin in mini Chinese cabbage (**Figure 2**), while exogenous BRs increased these cell wall components, thus maintaining the stability of the cell wall structure. The regulation of BRs on the content of cell wall components may be due to the signal cascade mediated by BRs to regulate the gene expression involved in the synthesis of cell wall biological components. For example, in Arabidopsis, the BR-activated transcription factor BZR1 and its homologous gene BZR2/BES1 have been shown to directly bind to promoter regions of a large number of cell wall-related genes (Jiang et al., 2015), including the majority of cellulose synthase genes (Xie et al., 2011). Wolf et al. (2012) reported that BRs signaling in Arabidopsis is coupled with the modification of methyl-esterified HGs to control pectin-dependent cell wall integrity (Wolf et al., 2012).

In the process of photosynthesis, solar energy, carbon dioxide, and water are utilized to produce oxygen and energy in the form of sugar, thus maintaining the life activities of the organism (Simkin et al., 2019). It is widely believed that the stronger the photosynthesis of a plant, the more organic matter is synthesized, and the more adequate the energy supply is, contributing to the robust growth of the crop and a higher final yield. The chlorophyll fluorescence parameters are important photosynthesis indicators and reflect the effect of stress on the photosystem (Deell et al., 1999). Tip-burn is also a physiological disease of mini Chinese cabbage caused by Ca^2+^ deficiency stress. As our experimental results showed, under Ca^2+^ deficiency treatment, chlorophyll fluorescence parameters (Fv/Fm, NPQ, and qP) of mini Chinese cabbage seedlings decreased significantly, while exogenous BRs application alleviated these effects and restored the chlorophyll fluorescence parameters to near normal levels (**Table 2**). Our results are basically consistent with the research results of Zhao et al. (2017), who found that application of exogenous EBR reversed the suppressive effects on photosynthesis, antioxidant enzyme activity, Rubisco activase (RCA), and gene expression induced by combined drought and heat stress in common wheat. Exogenous EBR can mitigate the adverse effects of high temperatures on plant growth by improving the maximum quantum yield of leaves, the excitation capture efficiency of PSII centers, and the effective quantum yield of photochemistry (Zhang et al., 2013). The photochemical quenching coefficient reflects the redox state of the primary electron acceptor and the number of open centers in photosystem II. The larger the qP, the greater the amount of QA reoxidation to form QA, indicating greater PSII electron transfer activity (Deell et al., 1999). The differences in chlorophyll fluorescence properties of plant leaves also reflected the differences in their photosynthetic capacity. Photosynthetic gas exchange parameters are the basis for measuring the exchange between plants and the outside world, and are the indicators of photosynthetic intensity. Lawlor (2002) reported that since CO_2_ enters leaves through stomata, the change of stomatal opening is very important for the study of photosynthesis. BRs can improve the drought tolerance of maize by improving the photosynthetic gas exchange parameters (Anjum et al., 2011). BRs are also able to promote photosynthesis and growth by positively regulating the synthesis and activity of several photosynthetic enzymes in cucumber, including Rubisco, as well as increasing photosynthesis by regulating the expression of *P450* and *MRP* (Xia et al., 2009), these results are basically consistent with our results. In our present study, calcium deficiency decreased stomatal conductance but increased the accumulation of intercellular CO_2_, suggesting that non-stomatal factor was the main reason for reduced photosynthesis caused by calcium deficiency. At the same time, BRs increased the activity of Rubisco and FBPase in Ca^2+^ deficiency treated leaves (**Figure 3**). The above results suggested that the application of exogenous BRs effectively mitigated the damage degree of photosynthesis in mini Chinese cabbage caused by calcium deficiency.

When plants are subjected to stress, chloroplasts are affected to some extent, with changes in their shape, detachment from the cell membrane, disappearance of starch granules, increases in osmiophilic granules, vesicle expansion, loosening and distortion, and significant deformation of palisade and spongy tissues. Exogenous phytohormone treatment causes chloroplasts in plant leaves to exhibit an intact internal lamellar system with normal chloroplast shape, intact cell membrane structure, and reduced osmiophilic granules, and mitigates cell structural damage and destruction (Meng et al., 2014). This is basically consistent with our findings, which Ca^2+^ deficiency-induced tip-burn in mini Chinese cabbage resulted in reduced numbers of cellular chloroplasts and grana, disorganized stromal lamellae, stromal wall separation, increased osmiophilic granules, lack of chloroplast adherence to the cell wall, and blurred chloroplast membrane structure, with some chloroplasts even differentiating into irregular structures. In contrast, exogenous BRs spraying reduced the aforementioned cell damage, leaving the cell membrane structure intact and the chloroplast structure relatively intact, and thus maintaining a relatively good vesicle-like structure (**Figure 4**). These findings demonstrated that the application of exogenous BRs mitigated calcium deficiency induced tip-burn by improving photosynthesis *via* increasing the activities of Rubisco and FBPase and protecting the leaf mesophyll cells and chloroplast structure of mini Chinese cabbage.

Photosynthetic pigments are the important material basis of leaf photosynthesis: Chla is the photosynthetic pigment for energy conversion, and Chlb and Car capture and transmit light energy. As shown in **Figure 5**, calcium deficiency significantly reduced the Chla and Chlb content in mini Chinese cabbage leaves, while exogenous BRs increased the content of photosynthetic pigments and somewhat alleviate calcium deficiency -induced attenuation of photosynthesis. These findings are consistent with those of the study by Yang et al. (2018), who reported that the application of BRs on leaves could promote the photosynthesis and chlorophyll fluorescence characteristics of *Leymus chinensis* under varying levels of shade. During leaf senescence, chlorophyll is removed from thylakoid membranes and converted in a multistep pathway to colorless breakdown products that are stored in vacuoles, and removal of Mg to form pheophytin is likely the first step, followed by removal of the phytol tail, catalyzed by pheophytinase (PPH), production of amphetamine (Eckardt, 2009). PPH, a chloroplast-located and senescence-induced hydrolase, is widely distributed in algae and land plants. In the process of plant senescence, *PPH1* gene can activate PPH and accumulate pheophytin (Schelbert et al., 2009). Pheophorbide a oxygenase (PAO) activity is found only during senescence, hence PAO seems to be a key regulator of chl catabolism (Pružinská et al., 2003), which is positively correlated with senescence. In our experiments, exogenous BRs application increased chlorophyll content, enhanced photosynthesis, and promoted the return to normal growth of mini Chinese cabbage by down-regulating the expression of *BrPPH1* and *BrPAO1* genes related to chlorophyll degradation in mini Chinese cabbage leaves (**Figure 6**). These findings are largely consistent with those presented by Liu et al. (2016). They found that BRs antagonistically regulated rice flag leaf senescence by mediating chlorophyll degradation, membrane degeneration, and senescence-related gene expression. These findings suggested that the application of exogenous BRs mitigated calcium deficiency induced tip-burn by maintaining a higher chlorophyll content *via* down-regulating the expression of chlorophyll degradation related genes, finally delay the leaf senescence caused by calcium deficiency.

## Conclusion

Exogenous BRs alleviated calcium deficiency induced tip-burn *via* 1): maintaining cell wall structure stability by increasing the contents of cell wall components, 2) improving photosynthesis by increasing the activities of Rubisco and FBPase enzymes, maintaining a relatively complete chloroplast structure and 3) as well as maintaining a higher chlorophyll content by down-regulating the expression of chlorophyll degradation related genes. Our results will provide theoretical basis and technical reference for prevention strategies of tip-burn in mini Chinese cabbage in future.

## Data availability statement

The original contributions presented in the study are included in the article/supplementary material. Further inquiries can be directed to the corresponding authors.

## Author contributions

YL: methodology, validation, formal analysis, writing-original draft, and writing - review & editing. JM and XG: writing - review & editing. JT, YW, and ZT: analyzed the data and prepared the figures and illustrations. JY: funding acquisition. LH: methodology, conceptualization, funding acquisition, and writing - review & editing. All authors read and approved the submission of the manuscript.
